# Will it Float? Rising and Settling Velocities of Common Macroplastic Foils

**DOI:** 10.1021/acsestwater.1c00467

**Published:** 2022-05-17

**Authors:** Boaz Kuizenga, Tim van Emmerik, Kryss Waldschläger, Merel Kooi

**Affiliations:** † Wageningen University and Research, Hydrology and Quantitative Water Management Group, 6700 AA Wageningen, The Netherlands; ‡ Wageningen University and Research, Aquatic Ecology and Water Quality Group, 6700 AA Wageningen, The Netherlands

**Keywords:** environmental fluid mechanics, experimental, marine debris, plastic pollution, microplastic, hydrology, hydrodynamics

## Abstract

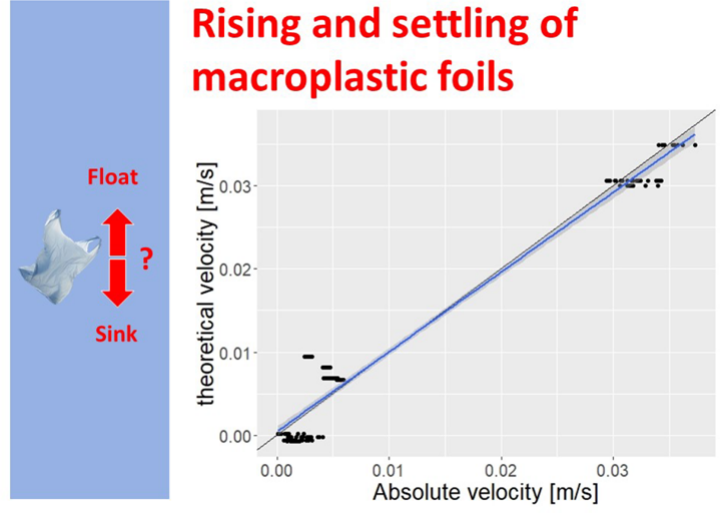

Plastic accumulates in the environment because of insufficient waste handling and its high durability. Better understanding of plastic behavior in the aquatic environment is needed to estimate transport and accumulation, which can be used for monitoring, prevention, and reduction strategies. Plastic transport models benefit from accurate description of particle characteristics, such as rising and settling velocities. For macroplastics (>0.5 cm), these are however still scarce. In this study, the rising and settling behavior of three different polymer types (PET, PP, and PE) was investigated. The plastic particles were foils of different surface areas and shapes. The observational data were used to test the performance of four models, including one developed in this study, to estimate the rising/settling velocity on the basis of the plastic particle characteristics. These models are validated using the data generated in this research, and data from another study. From the models that were discussed, the best results are from the newly introduced foil velocity model (*R*^2^ = 0.96 and 0.29, for both data sets, respectively). The results of our paper can be used to further explore the vertical distribution of plastics in rivers, lakes, and oceans, which is crucial to optimize future plastic monitoring and reduction efforts.

## Abstract 2

This paper contributes to a better understanding of vertical macroplastic transport, which is crucial for fate modeling and designing clean-up efforts.

## Introduction

Plastics are highly durable, are lightweight, and are cheap to manufacture, which makes them a popular resource for a variety of (single-use) products. Because of the high durability, they do not decompose easily and stay in the environment for a long time. This results in an accumulation of plastic waste in the environment, such as terrestrial, riverine, and marine ecosystems.^1−4^

Rivers transport land-based plastic toward the sea, and plastic pollution causes environmental damage to the river ecosystems and human livelihood.^3,5^ To manage and prevent the plastic waste streams in rivers, it is necessary to better understand their behavior in freshwater. More specifically, little is known about the vertical distribution of macroplastics below the surface. A theoretical approach to estimate the vertical distribution of plastics will complement and improve the development of observation-based methods, for example, new monitoring techniques, empirical methods, and other approaches for under water plastic estimates.^6,7^

Rising and settling velocities of plastic items and particles are crucial variables that determine the vertical movement of plastics. The terminal velocity of particles is one of the main parameters when it comes to sedimentation models.^8^ Knowing the terminal rising and settling velocities allows for a better selection of plastic collection technologies,^9^ which may depend on the vertical distribution of plastics. The vertical velocities depend on the properties of the plastics and determine the fate of the particles. Therefore, a better understanding is needed to predict how particles move in water and where settling hot-spots will occur.

Most research that was done on the rising and settling velocities focused on microplastics (plastics with a diameter ≤5 mm) in salt water.^10−14^ Some research has been conducted on rising and settling velocities of microplastics in fresh water,^15,16^ but there is no systematic research on settling and rising of macroplastics. The research that is done on macroplastics (plastics with a diameter >5 mm) in fresh water^17^ focused on plastic collected from the environment and did not consider different shapes and surface areas of the same polymers. Therefore, a systematic analysis of rising and settling velocities of macroplastic in fresh water is needed to gain a better understanding of the plastic transport in natural systems.

Here, we systematically performed rising and settling velocity measurements on foils (a minimum thickness/length/width ratio of 1:16:16^18^) for three different polymers. Foils were selected as this shape is only rarely addressed in current research^19^ and because they are a common shape in the environment.^20^ Furthermore, four different models that calculate the theoretical velocity in dependence of the particle properties were reviewed on the basis of this data set and the data set of Waldschläger et al.^17^ Three of these models are from the literature^21−23^ and one was newly developed.

Every model is different, but they are all based on the same characteristics of the particles and fluid: fluid density and particle properties such as material density, shape, and diameter. Foils behave differently than more spherical particles, and it is therefore unclear whether these models are suitable to estimate rising and settling velocities for macroplastic foils.^19^ With this paper we present (1) a laboratory method to perform macroplastic rising and settling velocity measurements and (2) a new model to theoretically determine the velocity based on the item characteristics.

## Materials and Methods

Three different polymer types were systematically researched on their rising or settling velocity. Furthermore, four different models were tested on their ability to estimate the rising and settling velocity of the plastics.

### Plastic Item Selection

**Figure 1:**
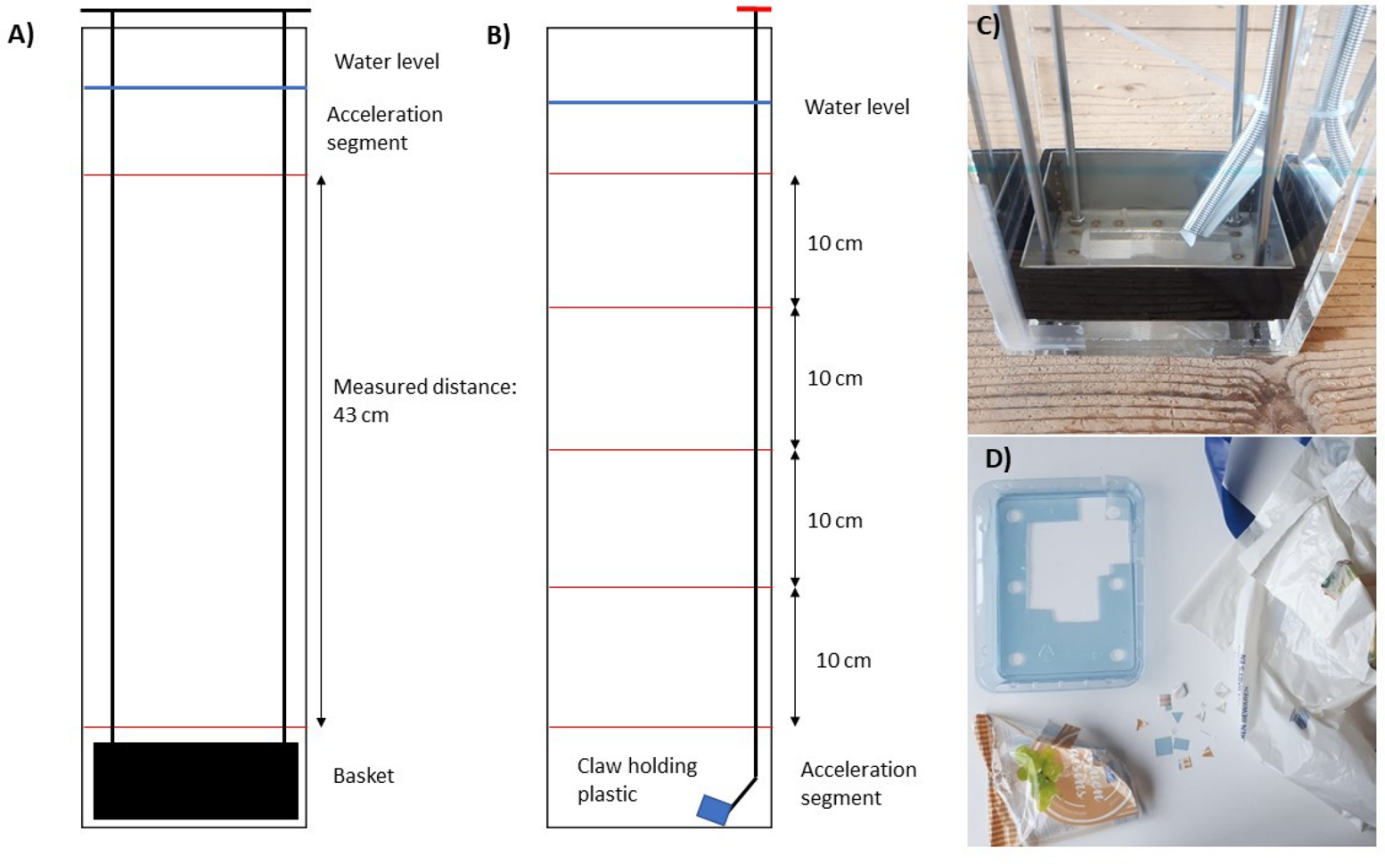
(A) Schematic setup for the settling velocity measurements. The red lines indicate the start and stop line for the stopwatch. The basket for retrieving the particles is visible at the bottom. (B) Schematic setup for the rising velocity measurements. The red lines indicate the start and stop lines for the stopwatch. (C) Close-up of the claw mechanism, which is holding a piece of plastic in place before measuring. (D) All sampled items for the experiments: the mushroom cover (PET) on the top left, the plastic bag (PE) on the right, and the raisin packaging (PP) on the bottom left.

In this study, we focused on the three most abundant plastic types found in the environment, namely, polyethylene terephthalate (PET), polypropylene (PP), and polyethylene (PE).^24^ Furthermore, plastic bags, food packaging, and PET items such as bottles are very common in the environment.^25,26^ PET has a density higher than water (1370 < ρ < 1450 kg/m ^3^(27)) and will therefore sink in natural, stagnant waters. PE and PP have densities lower than water (910 < ρ < 970 kg/m^3^ and 900 < ρ < 910 kg/m^3^, respectively^27^) and will therefore rise when submerged in a water column. The plastics were bought in the supermarket. For PET, the lid of a mushroom box was used, for PP, a raisin packaging, and, for PE, a shopping bag. These were manually cut in different shapes and sizes (Table 1 and Figure 1D) using a ruler and knife.

**Table 1:** Overview of Measurements That Were Carried Out^a^

material	surface area [cm^2^]	shape	L × W × H [cm]	# of measurements
PET	1.25	R	1 × 1.25 × 0.03	10
	1	R	1 × 1 × 0.03	10
	0.5	T	1 × 1 × 0.03	12
	0.25	R	0.5 × 0.5 × 0.03	10
PP	1.25	R	1 × 1.25 × 0.016	11
	1	R	1 × 1 × 0.016	10
	0.5	T	1 × 1 × 0.016	10
	0.25	R	0.5 × 0.5 × 0.016	11
	0.075	R	0.05 × 1.5 × 0.016	10
PE	1.25	R	1 × 1.25 × 0.004	10
	1	R	1 × 1 × 0.004	10
	0.5	T	1 × 1 × 0.004	10
	0.25	R	0.5 × 0.5 × 0.004	16
	0.075	R	0.05 × 1.5 × 0.004	10

a R = rectangle, T = triangle. PET is settling, and PE and PP are rising.

### Experiment Setup

The measurements were done in an acrylate column with a cross-section of 10 × 10 cm^2^ and a height of 70 cm (Figure 1A), filled with tap water of 15.6 °C. The water temperature was measured using a digital thermometer to calculate the viscosity. The particle sizes were chosen such that there would be no influence of the wall of the column on the measurements (the wall was not touched by the particle during the run). The settling and rising times of the plastics were recorded over a certain vertical length. A previous study, using similarly sized plastics, showed that plastics reach their terminal velocity within 15 cm.^17^ To be sure, the first 20 cm of the column was used for acceleration of the plastic in this research. This was done for both rising and settling velocity measurements.

#### Settling Velocity

The PET particles were released in the water column completely submerged, to make sure that no air bubbles were attached to the plastics and that they would not float because of the surface tension of the water. For the settling velocity measurements, a basket was put at the bottom to make it easier to pick up the particles after the measurements, and the same item could be measured repeatedly (Figure 1A). After the particles were retrieved from the water column, the basket was put back into the column. To make sure the water column was stagnant, the new measurements were only done if the water column appeared stable but at least after 1 min. A stopwatch was started when the particle reached the line 20 cm below the water surface. The bottom line—where the stopwatch was stopped—was placed at the lowest possible position, without having the basket interfere with the particles. This resulted in a distance of 43 cm over where the measurement was conducted (Figure 1A).

#### Rising Velocity

For the rising velocity measurements, the water column was divided in six areas (from the bottom up): an acceleration part of 20 cm, four measurement parts of each 10 cm, and the excess part. These four measurements per particle were only done for the rising velocity measurements (Figure 1B), because of the low rising velocity the particles have. Using this method, it allows for more measurements per particle without having to emerge the particle every time.

To make sure the measurements are done in a stable water column, a release mechanism at the bottom of the column is required for rise velocity measurements. Previous methods for releasing the plastics were too difficult for macroplastics or did not inquire a stagnant water column.^15,28^ That is why, for the rising velocity, a new method for releasing the particle was made. The new method consists of a flexible ‘claw’ mounted onto an aluminum frame (Figure 1C). The claw is held into a corner, making it possible to release the plastics without interfering the flow. By pushing on top of the claw, the hook releases the plastic without having to disturb the water. This way, the water remains as stagnant as possible.

First, a test run was done for the plastic to determine the position of the release mechanism and the time it takes for the plastic to reach the surface. Depending on this time, the distance over which the plastic was measured was chosen. The four 10 cm lines (Figure 1B) were taken together in either parts of 20 or 40 cm if the plastic was fast to make sure the measurements were precise. Measurements of 10 cm were chosen if the plastic was slow. So, if 10 cm was chosen then for one run the time was recorded four times.

### Model Evaluation

To estimate the rising and settling velocities of other plastics, mathematical models were used that estimate velocity using the size, shape, and density of the particle, and the properties of the water, such as viscosity and density, were taken into account. The dynamic viscosity was estimated using the measured temperature of the water. For all theoretical velocities, the density of water was estimated at 999 kg/m^3^ (for 15 °C). The densities of the plastics were obtained from Hidalgo-Ruz et al.^27^ From the range mentioned in the article, the mean was taken as a density for each polymer type.

To get a better view on the validity of the models, two data sets were used. One was the data set collected in this research, and the other was the data from Waldschläger et al.,^17^ which includes mainly microplastics of different shapes (particles with an equivalent diameter (, in which *a*, *b*, and *c* are the side lengths) ranging between 0.58 and 30.81 mm^17^).

Because some models make assumptions that are based on the turbulence of the flow, the Reynolds numbers (a measure for turbulence) for all polymers were calculated, using eq 1. This can give an indication of the applicability of the models.
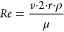
1Using R, a plot was made to show the relationship between the Reynolds number and the measured velocity (see the Supporting Information).

In eq 1, *r* is the equivalent sphere radius (ESR) of the particle in meters (unless stated otherwise), ρ the density of water in kilograms per meter cubed, μ the dynamic viscosity of water in pascals per second, and *v* the velocity of the particle in meters per second. The ESR is calculated using the volume of the particles, and relating that volume to a sphere. From there, the radius of that sphere is taken as *r*.

A theoretical settling velocity was calculated for all plastic items, given the parameters above and the plastic size and density. When these theoretical velocities and the measured data are plotted against each other, the points should lie on the line *y* = *x* (which is plotted in every graph), and an *R*^2^ was calculated with respect to *y* = *x* to evaluate the model performance. A *p*-value was calculated using the F-statistic *p*-value generated by R.

The four models that were reviewed are (1) the Stokes model for laminar flow,^23^ (2) a model based on both laminar and turbulent flow,^21^ (3) a settling velocity model based on the Hofmann shape entropy,^22,29,30^ and (4) a model based on the turbulent drag force, derived in this research.

These models base their velocity on a shape factor or on a constant that is empirically determined, in which the shape of the particle plays a role. This is relevant, because the particles measured in this research have a shape that only rarely is found in natural grains. Therefore, the value of these models for relatively flat particles and foils is researched. A summary of all models is given in Table 2.

**Table 2:** Summary of the Researched Velocity Models^a^

model	*Re* regime	*R*^2^ for *y* = *x* (1)	*p*-value (1)	*R*^2^ for *y* = *x* (2)	*p*-value (2)
Stokes	<1	–0.17	<2 × 10^–16^	–0.11	0.0162
Ferguson and Church	<100.000	+0.58	<2 × 10^–16^	–0.73	<2 × 10^–16^
Le Roux	<100.000	–0.99	<<2 × 10^–16^	–2 × 10^51^	0.465
FoMo with calibration	turbulent	0.96	<2 × 10^–16^	+0.29	<2 × 10^–16^
FoMo, no calibration	turbulent	–0.37	<2 × 10^–16^	–0.79	<2 × 10^–16^

a (1) is the dataset from this research and (2) is the dataset from Waldschläger et al.^17^.

The first model for settling velocity that was reviewed, was the Stokes equation for settling velocity (eq 2). Stokes derived this from the simplified Navier–Stokes equations. Although this relation can only be used for very low Reynolds numbers,^17^ the Stokes equation forms the basis for a lot of models for settling velocity of natural grains and was thoroughly studied. It can also be used for plastic, at least in an adjusted form.^21,31,32^
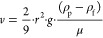
2In this equation, *g* is the gravitational acceleration in meters per second squared, μ the dynamic viscosity of water in pascals per second, and ρ_p_ and ρ_f_ are the densities of the particle and the fluid in kilograms per meter cubed, respectively. The more the particle shape deviates from a sphere, the worse the usability for the Stokes’ equation gets. That is why the Stokes equation works best for perfect spheres.

A different equation for settling velocity was developed by Ferguson and Church:^21^
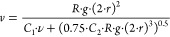
3In which  (submerged specific gravity), *r* is in centimeters, and *g* is the gravitational acceleration in meters per second squared. For the polymers with a density lower than water, the submerged specific gravity was taken absolute in the denominator, because of the power 0.5. The constants *C*_1_ (constant from Stokes’ law for laminar settling) and *C*_2_ (drag coefficient for Reynolds numbers exceeding 10^3^) are based on the shape of the particle and the properties of the fluid. The difference with the Stokes model is that this model incorporates a factor for turbulent flow and is therefore applicable at a larger range of Reynolds numbers.

For smooth spheres, *C*_1_ and *C*_2_ were determined to be 18 and 0.4, respectively, but for particles with other shapes, these values will become higher. In this research, values of 24 for *C*_1_ and 1.2 for *C*_2_ were assumed, as these are the theoretical limit for very angular grains for this model.^21^ Because this equation includes turbulent drag, it can be used for Reynolds numbers up to 100,000.^21^

A third theoretical approach is based on the Hofmann shape entropy (HSE, eq 4), which was formulated by Hofmann.^29^ The HSE is a shape factor that describes the shape of a particle, with 1 being a perfect sphere.
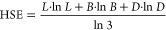
4

In eq 4, *L*, *B*, and *D* are the length, width, and thickness of the particle in meters, respectively.

According to Van Melkebeke et al.,^19^ no shape factor can differentiate between foils, fibers and granular particles, but a shape factor can be used to describe particles within a certain shape. The velocity model based on the HSE is mainly used for ellipsoid particles,^30^ but can also be used for irregular shaped grains.^22^ In this research, eq 5 was used, which was derived by Le Roux:^22^
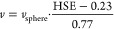
5In eq 5, *v*_sphere_ is the theoretical velocity (in meters per second) if the particle is a perfect sphere (which was derived in Le Roux^33^) and the constants are empirical. Because of the HSE and the constants, this model can be used for other shapes as well. This model can be used for *Re* < 100,000.^22,30^Equation 5 is the end product of this derivation.

The last equation that was used in this research, is named the foil velocity model (FoMo)(eq 11). This equation was derived within this study.

The FoMo follows from the idea that, when the gravity force (eq 6), buoyancy force (eq 7), and drag force (eq 8) are equal (eq 9), the particle reaches its terminal velocity.
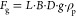
6
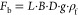
7
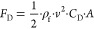
8
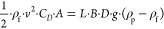
9
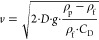
10

In the equations above, *C*_D_ is the drag force constant, ρ_f_ and ρ_p_ are the density of the fluid and the particle in kilograms per meter cubed, and *A* is the area of the particle in meters. According to Batchelor,^34^ the drag force constant can be assumed constant from *Re* = 3500 for well-defined spheres up to *Re* = 10^7^ for poorly defined shapes. In this research, we assume that the Reynolds’ number is sufficiently high to assume *C*_D_ constant and assume that the flow is turbulent.

It was observed that during the settling velocity experiment, the foils came down with a swaying, sideways motion. Because of this, it is assumed that the thickness *D* can better be approximated with the ESR (‘r’ in the equation) times the CSF, which is the shape factor defined by Corey^35^ and McNown and Malaika:^36^. Accourding to Francalanci et al.,^37^ this is the best shape factor for describing particle shape. This results in the final velocity model for foils:
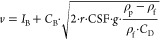
11In eq 11, *r* is the equivalent radius in meters, *g* is the gravitational acceleration in meters per second squared, ρ_f_ and ρ_p_ are the density of the fluid and the particle in kilograms per meter cubed, and *C*_B_ and *I*_B_ ([meters per second]) are empirical constants. The radius of the particles was calculated in the same way as for the other equations. The drag constant *C*_D_ was assumed at 1.5, because the particles are relatively flat and will thus have a lot of turbulent drag.^38^ For this equation, the measured velocity was transformed to an absolute velocity, since eq 11 can not model negative velocities because of the square root.

As this model was derived from theory, two empirical constants were introduced (*C*_B_ and *I*_B_) to make the best fit for this model. This calibration was done by performing a linear regression analysis. First, the constant *C*_B_ was assumed at 1, and *I*_B_ was assumed to be 0 (that is true if the model is perfect). After this, the model was corrected for the slope of the model with the old constants, using the regression result. By assigning new values for the constants, the model was changed to obtain a better fit with the measured data. The model was validated using the data from Waldschläger et al.^17^ In that study, for 100 particles collected from a fluvial environment, the rising or settling velocity was measured. The data set ranges from microplastic to small macroplastic particles of different polymer types. To see if the FoMo model is generally applicable to other data sets, this calibrationwas also done on the data set by Waldschläger et al,^17^ and then tested on the data from this research.

## Results and Discussion

In this study, settling and rising velocities of different flat plastic particles were measured, and four different models were fitted to the data.

PET was found to have a relatively large settling velocity (0.029–0.037 m/s). This could indicate that PET sinks to the bottom of a fresh-water system quite fast. However, the larger the PET foil is, the slower it will sink. PE and PP are found to rise relatively slow (0.0001–0.004 and 0.002–0.006 m/s, respectively). This might indicate that they are more likely to be distributed over the water column and that they are more influenced by turbulent movements in the river. The rising and settling velocities found may change with different ambient settings. For example, seawater will result in different velocities and model parameters. However, research is needed to know how the plastics will react to the different ambient densities.

Waldschläger et al.^17^ found rising velocities in the range 0.0016–0.0352 m/s and settling velocities in the range 0.0018–0.199 m/s. The ranges found in this research correspond to those velocities. The data of Zaat^28^ also corresponds to the data found in this study (0.021–0.009 m/s for rising velocity).

In Table 2, the results and assumptions of all the models are summarized. In contrast to other research on rising velocity of macroplastics,^13,28^ this research included a new method for the plastic release without disturbing the water column. This means that there are no influences of turbulent water flow in the column, and the results are reliable.

A lot of research on environmental plastics is done on microplastics,^12,13,15,16^ but to date, not much research has been done on macroplastics.^17,28^ Zaat^28^ performed measurements on large pieces of low and high density PE, but in these experiments, a stable column was not inquired.

The Reynolds number is a measure for turbulence (eq 1). The Reynolds regime of this experiment falls in the following range: 12 < *Re* < 10,000. The four models that were used in this study are valid for different Reynolds regimes (Table 2).^21−23^ Stokes equation gives only an inaccurate approximation, because that model is most suited for very low Reynolds numbers because of the assumptions made in the derivation.^23^ The other models do work for this regime and are therefore more suitable to be applied to the data.

**Figure 2:**
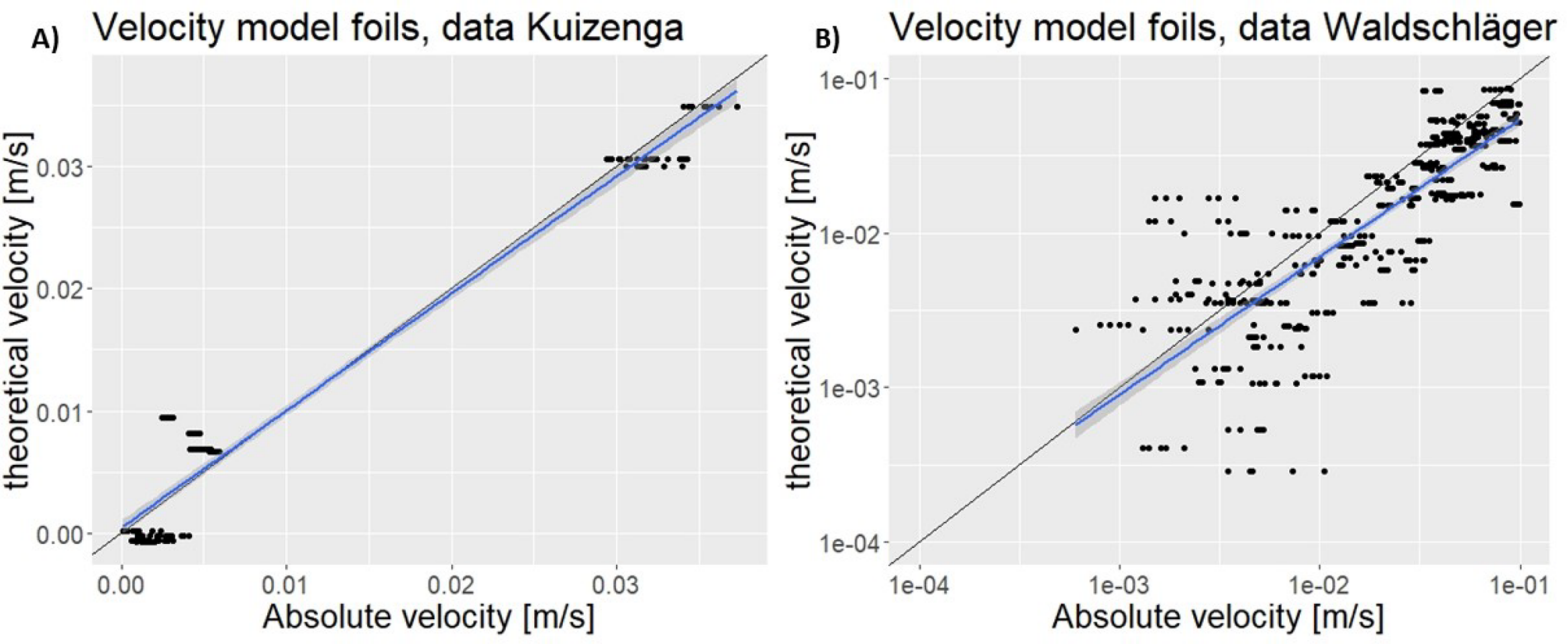
FoMo plotted with (A) the data generated in this research and (B) the data from Waldschläger et al.^17^. The gray area is the standard error. The line *y* = *x* is shown as a black line.

All models discussed were plotted against the measured velocities from the data sets. The plots for the models from literature are available in the Supporting Information; the plots for the FoMo are shown in Figure 2. The FoMo was calibrated with the data generated in this research and therefore responds best from all models on this data set. Two empirical constants were introduced to fit the data better, which have values of *C*_B_ = 1.96 and *I*_B_ = −0.004. Because eq 11 has a square root, the results of the rising velocity experiments were taken as absolute. This could give a different value for the constants *C*_B_ and *I*_B_. The FoMo also performs best on the data set by Waldschläger et al.,^17^ which is a data set based on different particle types and sizes, without adjusting the parameters.

When the constants arere calibrated on the data set by Waldschläger et al.,^17^ the FoMo still gives the best results compared to the other models (Tables 2 and 3). However, because the data set of Waldschläger et al.^17^ consists of various plastic types and shapes, the estimate becomes worse. The constants found when the calibration is done on Waldschläger et al.^17^ are *C*_B_ = 1.904 and *I*_B_ = 0.007.

**Table 3:** Results from the Model Evaluation When the Constants Are Calibrated on the Dataset by Waldschläger et al.^17^

*R*^2^ for different calibrations	calibrated on Kuizenga	calibrated on Waldschläger
data set Kuizenga	0.96	0.21
data set Waldschläger	0.29	0.34

In Waldschläger and Schüttrumpf,^15^ six models from sedimentation theory were researched for microplastics. The Stokes model was also researched in that model, because it is still the most commonly used model, but the others are different. In Waldschläger and Schüttrumpf,^15^ the models are found to estimate the behavior of all particles with insufficient precision. The same was found for the models from the literature in this research, on the basis of the data for macroplastics. The new model from this research shows promising results and should be researched further.

Van Melkebeke et al.^19^ researched different shape factors on their ability to describe different plastic shapes. They found that no shape factor is able to describe all different kinds of particles, and therefore, no model in this research would be able to describe all sorts of plastic. However, the FoMo performed relatively well on the data set from Waldschläger et al.,^17^ which included various plastic types and shapes.

A remark should be made on the measurements: the plastics were—in contrary to nature—not in water for at least a few hours before the velocity was measured. The exposure to water has a large impact on the rising and settling velocity of microplastics;^10^ however, the impact on macroplastics has not yet been determined. Furthermore, in the environment, biofouling and particle aggregation will take place, which will change the behavior of the plastics even further.^19,39^

The plastic densities in this research are from the literature. Although other studies also use densities from the literature (e.g., Kaiser et al.^40^), further research should measure the densities more precise. Density is a key parameter in the models, and if the plastic densities deviate from the densities found in the literature, this can result in model errors.

Future research can make use of particle image velocimetry (PIV) to measure larger speeds or multiple particles at once. For this research, a stopwatch was chosen to make the research easily duplicable and because the chosen particles were very slow.

Our systematic laboratory research on macroplastic can be used as a basis for further research on macroplastics in the environment. The use of models is a valuable aspect of this research, and—if researched further—can contribute to a better understanding of the behavior of plastics in the aquatic environment. Future research can be based on this study but should be elaborated. For example, more measurements with different plastics items, polymers, and shapes and experiments in flowing water and different flow regimes can improve the performance and transferability of the models.

## Conclusion

In this research, three different polymer types and five different surface area classes were tested on their rising and settling behavior. Three different models from the literature and one model derived from theory were used to calculate the velocity. The newly developed technique to release the macroplastics with a density lower than water (i.e., the rising plastics) worked. This method, consisting of a claw and an aluminum frame, is easy to use, allowing for reproducible experiments.

From all four models that were introduced, only two estimated the behavior of the flat particles relatively well-based on the measured data: the model by Ferguson and Church^21^ (*R*^2^ = 0.58) and the model based on the drag force that was introduced in this research (*R*^2^ = 0.96). All other models performed less when the data from Waldschläger et al.^17^ were used, compared to the data generated in this research. This is probably due to the bigger differences in shapes and sizes in the data from Waldschläger et al.,^17^ which models cannot accurately capture. Still, the data generated and model analysis performed in this study are valuable for further plastic research.

With this paper, we aim to shed new light on rising and settling velocities of common macroplastic items. We provide an experimental setup that can be used for future research and developed a simple model to estimate velocities on the basis of item characteristics.

## Supporting Information Available

Figures of models based on the newly generated data and Reynolds number plotted against the velocity measured (PDF)
