# Supplementary material for: Will it Float? Rising and Settling Velocities of Common Macroplastic Foils
Source: ACS ES T Water. 2022 May 17;2(6):975–81. doi: 10.1021/acsestwater.1c00467 (PMC9194906; doi:10.1021/acsestwater.1c00467)
Supplement: Supplementary file 1 — ew1c00467_si_001.pdf [file ew1c00467_si_001.pdf]

Supplementary material: Will it float? Rising and settling velocities of common macroplastic foils

Boaz Kuizenga,*,† Tim van Emmerik,† Kryss Waldschläger,† and Merel Kooi‡

†*Wageningen University and Research, Hydrology and Quantitative Water Management Group, Wageningen, The Netherlands*

‡*Wageningen University and Research, Aquatic Ecology and Water Quality Group, Wageningen, The Netherlands*

E-mail: boazkuizenga@gmail.com

Supplementary Data

The supplementary data (the dataset generated for this study) can be found at the 4TU.researchdata library under DOI: 10.4121/14709360.

Supplementary Tables and Figures

Figures

The figures can be found on the last page.

References

(1) Ferguson, R. I.; Church, M. A simple universal equation for grain settling velocity. *Journal of Sedimentary Research* **2004**, *74*, 933–937.

(2) Le Roux, J. P. Application of the Hofmann shape entropy to determine the settling velocity of irregular, semi-ellipsoidal grains. *Sedimentary Geology* **2002**, *149*, 237–243.

(3) Waldschläger, K.; Born, M.; Cowger, W.; Gray, A.; Schüttrumpf, H. Settling and rising velocities of environmentally weathered micro- and macroplastic particles. *Environmental Research* **2020**, *191*.

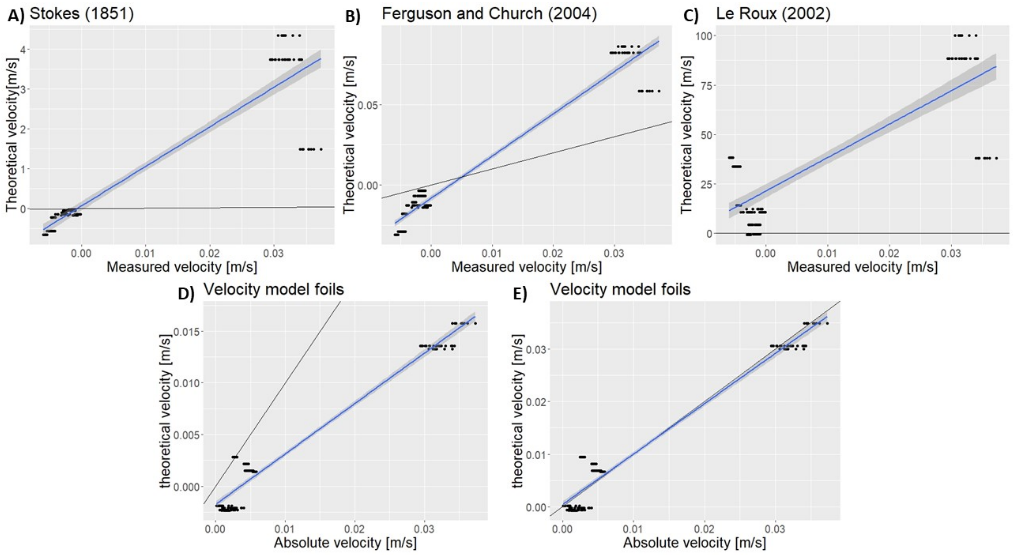

(a) Figures that show the models based on the newly generated data. A) is the Stokes' model, B) is the model by Ferguson and Church[1], C) is the model by Le Roux[2], D) is the velocity model for foils without constants, and E) is the velocity model for foils with fitted constants.

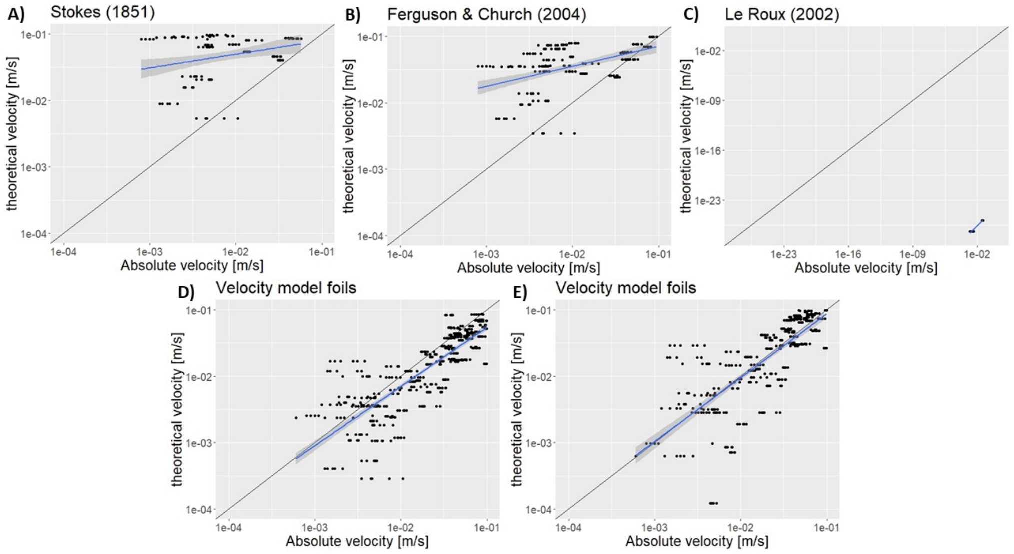

(b) Figures that show the models based on the data by Waldschläger et al.[3]. A) is the Stokes' model, B) is the model by Ferguson and Church[1], C) is the model by Le Roux[2], D) is the velocity model for Foils without constants, and E) is the velocity model for foils with constants fitted on the new data.

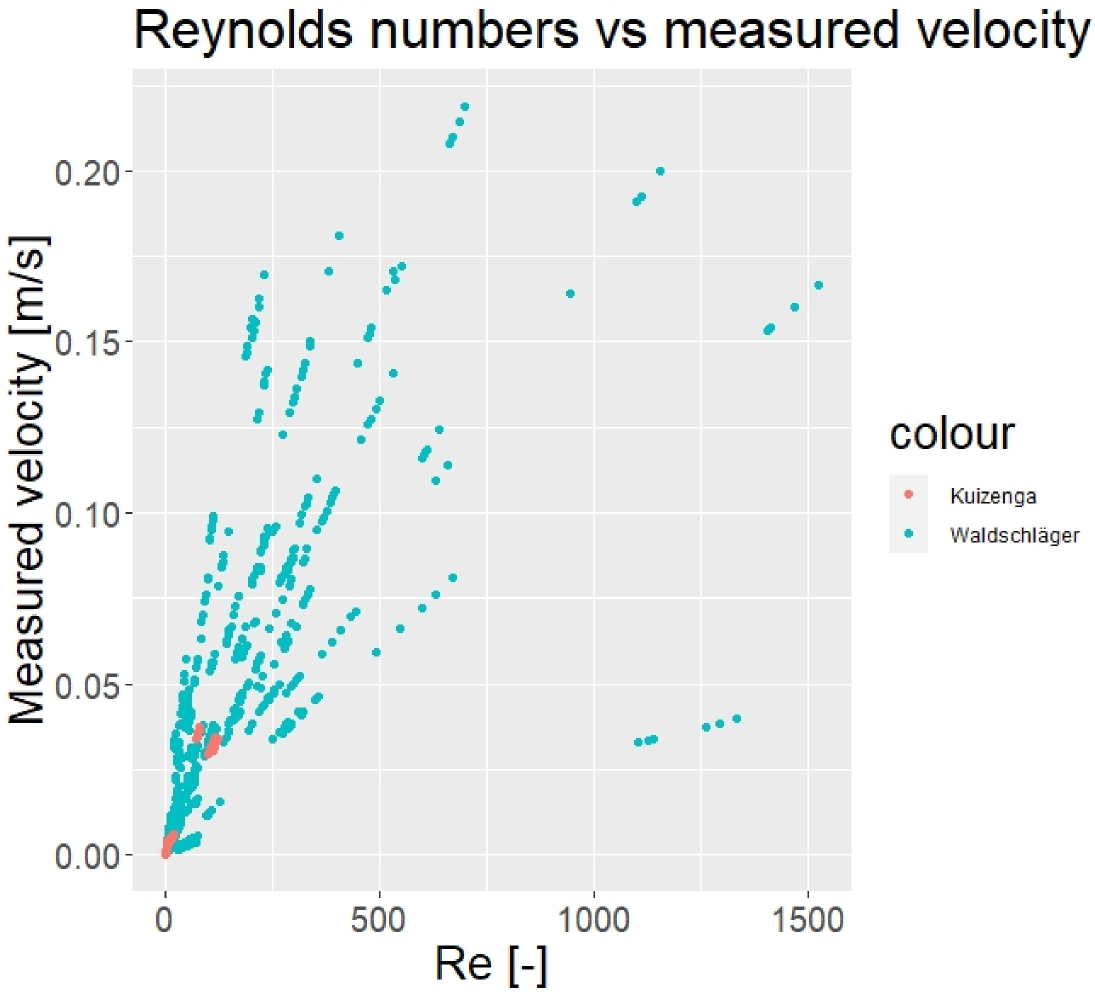

Figure 2: The Reynolds number plotted against the velocity measured. The blue datapoints are from Waldschläger et al.[3], the red datapoints are from this research.
